# Ranolazine: impact on quality of life in patients with stable angina pectoris, results from an observational study in Austria – the ARETHA AT study

**DOI:** 10.1007/s00508-019-1481-x

**Published:** 2019-04-08

**Authors:** Robert Zweiker, Josef Aichinger, Bernhard Metzler, Irene Lang, Eva Wallner, Georg Delle-Karth

**Affiliations:** 1 Department for Cardiology, Medical University Graz, Auenbruggerplatz 15, 8036 Graz, Austria; 2 2nd Internal Department, Hospital of the Order of St. Elisabeth Linz, Fadinger Straße 1, 4010 Linz, Austria; 3 University Clinic for Internal Medicine, Department for Cardiology, Medical University Innsbruck, Anichstraße 35, 6020 Innsbruck, Austria; 4 Cardiology, Medical University of Vienna, Währinger Gürtel 18–20, 1090 Vienna, Austria; 5 A. Menarini Pharma GmbH, Pottendorfer Straße 25–27, 1120 Vienna, Austria; 6 Department for Cardiology, Hospital Hietzing, Wolkersbergenstraße 1, 1130 Vienna, Austria; 7 Medical University of Vienna, Vienna, Austria

**Keywords:** Coronary artery disease, Clinical decision making, Drug prescriptions, Quality of life, Antianginal drug

## Abstract

**Objective:**

Assessment of treatment routine and outcome for ranolazine in clinical practice as second-line treatment for stable angina pectoris (AP).

**Design and setting:**

Multicenter, prospective, uncontrolled, non-interventional study at 88 sites including internal specialists, cardiologists, pneumologists, angiologists and primary care practices in Austria.

**Participants:**

In this study 292 patients receiving ranolazine in the course of routine treatment on top of beta blockers or calcium channel blockers after failure of first-line therapy.

**Main outcome measures:**

Dosage and symptoms were recorded at two visits (at intervals of 12 weeks), complemented by treatment rationale and disease characteristics at baseline. Disease intensity was quantified by angina symptoms, nitrate use and by Canadian Cardiovascular Society (CCS) grading. Quality of life (QoL) was assessed through a 10-grade scale. Data were analyzed by descriptive statistics.

**Results:**

Ranolazine was prescribed in order to improve exercise capacity (84.3%), reduce symptoms (83.2%) and reduce AP (77.1%). Of the patients 87.3% received the recommended starting dose of 375 mg and subsequent dose changes were reported for 39.8%. The number of AP attacks was reduced from 5.3 ± 4.5 to 0.8 ± 1.3 per week; nitrate use was reduced from 3.4 ± 4.1 to 0.4 ± 0.9 applications per week. Of the patients 94.0% reported improved exercise capacity and 93.7% reduced symptoms. For the majority of patients, the CCS improved from grade II to I and QoL improved accordingly. Of the patients 3 experienced adverse drug reactions and 95.5% continued ranolazine.

**Conclusion:**

In this real-world study, ranolazine was shown to be effective, safe and well tolerable. Symptoms of AP were improved, as illustrated by the reduced number of angina attacks, reduced rate of nitrate use, reduced CCS scores and improved QoL.

## Introduction

Coronary heart disease (CHD) is a major cause of death in the European population [1]. Treatment of CHD aims at the improvement of mortality and morbidity and at the mitigation of angina symptoms. In addition to treatment improving the prognosis, current guidelines of the European Society of Cardiology (ESC) recommend drugs for symptom control, including beta blockers, calcium channel blockers (CCB) and sublingual nitroglycerin for chest pain management [2]. In cases of failure of the first-line therapy, a switch to the other options (CCB or beta blocker), a combination of both, or the use of other antianginal drugs are to be considered. Second-line drugs can be used as first-line treatment in cases of intolerance or contraindications to both betablockers and CCBs.

This article presents real-life experience on the second-line antianginal drug ranolazine, a piperazine derivative approved for the treatment of chronic stable AP in 2008. Ranolazine causes a reduction in sodium and calcium overload, thus exhibiting antianginal effects by inhibition of the late sodium current in cardiac cells. Ranolazine is therefore expected to improve diastolic tone and oxygen handling during myocardial ischemia [3]. The place in treatment, general properties and guidance for treatment of stable angina is also outlined in the current ESC guidelines [4]. Ranolazine is indicated as an add-on for the symptomatic treatment of stable AP in adult patients who are inadequately controlled or intolerant to first-line antianginal treatment [2]. In Austria, which did not contribute to the pivotal ranolazine studies, failure and/or contraindication or intolerance towards CCBs and beta blockers are required to justify reimbursement of ranolazine by public health insurances [5]. According to registry data, ranolazine is used in up to 20% of the patients with stable coronary artery disease [6].

This non-interventional study ARETHA AT aimed to improve understanding of the use of ranolazine for the treatment of symptoms in patients with stable AP in routine clinical practice in Austria. Indications for the use of ranolazine are reported, and the findings are put in relation with the results of previous clinical and observational studies. The primary research focus was an evaluation of dose prescription at the screening visit as well as any changes during the course of the study of approximately 12 weeks. The secondary research interests included (1) dose changes, (2) clinical condition for ranolazine prescription including previous therapies, (3) quality of life (QoL), (4) response to ranolazine treatment, and (5) safety and tolerability based on adverse drug reactions and vital signs.

## Patients, materials and methods

### Design and conduct of the study

The ARETHA AT was a non-interventional, non-randomized, uncontrolled post-marketing observational study conducted in a prospective, single country, multicenter format, approved by the responsible independent ethics committee under the code of 27-232 ex 14/15. A screening visit (visit 1) and a subsequent final examination visit (visit 2) approximately 12 weeks later were scheduled at the physician’s discretion. This treatment regimen is required for the reimbursement of treatment costs by social insurance [5], although no visit dates were specified by the protocol due to the non-interventional nature of the study. A total of 91 investigators from 88 doctors’ offices, including selected primary care doctors and medical specialists (internal specialists, cardiologists, pneumologists and angiologists) were involved. The selection of practices aimed at a balanced representation of health care facilities in Austria. The study was open to a maximum of 720 patients.

Prior to enrolment, patients were informed about the study and data evaluation. Inclusion was clearly separated from all therapeutic and diagnostic procedures, which were carried out according to standard medical care. Open-label ranolazine (sustained release tablets, 375 mg, 500 mg or 750 mg) was prescribed according to the summary of product characteristics (SmPC), which recommend an oral dose of 375 mg twice daily. After 2–4 weeks, the dose should be titrated to 500 mg twice daily and subsequently, according to the patient’s response, to a recommended maximum dose of 750 mg twice daily. Adverse events (AEs), such as dizziness, nausea and vomiting may require discontinuation or down-titration of ranolazine to 500 mg or 375 mg twice daily.

All subjects treated with ranolazine according to the SmPC were eligible except for the following exclusion criteria: (1) contraindications according to the SmPC, (2) pregnancy or lactation, (3) use of metformin during the study exceeding 1000 mg/day, (4) ranolazine treatment for more than 2–4 weeks prior to visit 1, (5) use of over 20 mg daily dose of simvastatin is a contraindication for ranolazine according to the SmPC. Any other concomitant therapies were permissible at the discretion of the treating physician.

The following variables were recorded and analyzed: (1) basic demographic features, (2) date and method of initial diagnosis of coronary artery disease (CAD), (3) risk factors, (4) physical activity, (5) exposure to stress and known trigger factors, (6) blood pressure and heart rate measured according to local routine, (7) disease activity according to the Canadian Cardiovascular Score (CCS), (8) QoL by Likert scale [7] recorded separately by physicians and patients, (9) comorbidities, (10) concomitant medications, (11) ranolazine therapy including dose changes and continuation decisions, (12) AE recording.

### Assessment of safety and tolerability

Any medically undesirable experiences including abnormal laboratory data, which occurred after the administration of ranolazine were considered as AEs. If the relationship to ranolazine was not classified as unrelated by the respective investigator, the AE was regarded as an adverse drug reaction (ADR). The AEs and ADRs were classified as severe if they resulted in death, were life-threatening, required or prolonged hospitalization, in persistent or significant disabilities or incapacities, led to congenital anomalies or birth defects or consisted of another medically important condition. Furthermore, ADRs were classified with respect to the intensity as mild, moderate or severe. All AEs were documented and reported in accordance with all applicable legislation and regulation but only ADRs, coded by Medical Dictionary for Regulatory Activities (MedDRA, www.meddra.org), were considered in this study.

### Statistical methods

Due to the observational nature of this study, no formal calculation of sample size was carried out. The maximum number of 720 participants was defined for organizational reasons and is not related to any scheduled statistical procedures. Statistical analyses were carried out using the analysis software MedCalc® 16.8.4 (MedCalc Software, Ostend, Belgium) and Microsoft Excel® 2010. The only analysis set comprised all eligible patients who took at least one dose of the study medication and for whom at least partial data were documented. Data were analyzed by means of descriptive statistics, including absolute and relative frequencies, sample size (*n*), minimum (min), maximum (max), arithmetic mean (µ), standard deviation (SD) and median. Subgroups were formed post hoc by patient characteristics and compared in an exploratory fashion. In the running text, continuous variables are characterized by arithmetic mean ± SD. Where appropriate, the Wilcoxon signed rank test was used to test selected changes from visit 1 to visit 2 for significance. All data were analyzed as observed without imputation of missing values. Concomitant medication was summarized by the Anatomical Therapeutic Chemical Classification System, level 2 (ATC2).

## Results

### Demographics and medical history

A total of 292 persons participated in visit 1 and 289 (99.0%) in visit 2. Of the patients 162 (55.5%) were male, mean age was 73.6 ± 10.6 years. Table 1 shows selected demographic and baseline characteristics. Of the patients 123 (42.1%) had received a diagnosis <1 year prior to inclusion. The main methods used for diagnosis included patient history (242; 82.9%), stress electrocardiogram (ECG, 182, 62.3%), coronary angiography (172, 58.9%), clinical signs (157, 53.8%), ECG (142, 48.6%) and myocardial scintigraphy (43, 14.7%). Thus, for most patients several methods had been used. Any use of coronary angiography, not necessarily related to first diagnosis, was reported by 257 patients (88.0%), in 167 cases (57.2%) including revascularization. Risk factors included a history of smoking (96 patients; 32.9%), hypertension (243; 83.2%), hyperlipidemia (225; 77.1%), obesity (117; 40.1%), diabetes mellitus (92; 31.5%) and family disposition (64; 21.9%). Comorbidities such as heart failure (94; 32.2%), arrhythmia (91; 31.2%), chronic kidney disease (68; 23.3%), depression (66; 22.6%) and hyperuricemia (46; 15.8%) were common.

**Table 1:** Demographic and baseline features of the participants

Total number of participants (*n*; %)	292; 100.0%
Male participants (*n*; %)	162; 55.5%
Age (µ ± SD; min–max)	73.6 ± 10.6; 24–96 years
Weight (µ ± SD; min–max)	79.9 ± 13.7; 49–125 kg
Height (µ ± SD; min–max)	169.2 ± 8.6; 147–190 cm
*Smoking behavior (n; %)*	
Smoker	41; 14.0%
Former smoker	55; 18.8%
Non-smoker	196; 67.1%
*Time since diagnosis (n; %)*	
<1 year	123; 42.1%
1–4 years	70; 24.0%
4–7 years	35; 12.0%
≥7 years	58; 19.9%
Revascularization	167; 57.2%
Diabetes	92; 31.5%
Hypertension	243; 83.2%
Hyperlipidemia	225; 77.1%

Of the patients 147 (50.3%) reported to be physically inactive; 100 patients (34.2%) performed exercise at least twice a week. Walking and hiking were the most frequently reported exercises (133; 45.5%), followed by endurance training (13; 4.5%). All other kinds of training were reported by less than 5% of patients. Of the patients 231 (79.1%) provided an estimate of their individual stress level on a 1–10 scale, reporting an average level of 4.7 ± 2.3.

Information on known triggers of AP attacks were available for all patients. Physical stress was reported most frequently (270; 92.5%), followed by mental stress (129; 44.2%), changing weather (77; 26.4%) and exposure to cold or wind (68; 23.3%). Other triggers occurred in <10%.

### Substance exposure

Ranolazine was administered according to therapeutic needs, whereby an expected improvement of exercise tolerance in everyday life (84.2%) and a reduction of angina (83.2%) were the most common reasons. Other treatment objectives included anginal attacks despite antianginal premedication (77.1%), reduction of fast-acting nitroglycerin preparations (28.8%) and the impossibility of revascularization (30.8%); a full list is shown in Supplement Table 1. Of the patients 281 patients (96.2%) received ranolazine twice daily, 10 patients (3.4%) once daily, no dosing information was available in 1 case, 254 patients (87.3% of those with data available) received the recommended starting dose of 375 mg, whereas 37 patients (12.7%) started with a higher dose compared to the SmPC. During the course of the study, any kind of dose change was reported for 116 patients (40.7% of those with data available), while 169 patients (59.3%) continued the dose prescribed at baseline. Among those patients for whom ranolazine was prescribed after the completion of the study, 132 (50.0%) received a dose of 375 mg according to the physician’s judgement (Table 2), including cases of repeated dose changes. After visit 2, 276 patients (95.5%) continued and 13 patients (4.5%) discontinued treatment. Reasons for discontinuation included lack of efficacy in four cases, and adverse events in three cases. In six patients, discontinuation occurred for other reasons.

**Table 2:** Dosing of ranolazine at visit 1, visit 2 and after the study

Dosage	Visit 1 *n* (%)	Visit 2 *n* (%)	Future use *n* (%)
375 mg	254 (87.3)	133 (49.1)	132 (50.0)
500 mg	34 (11.7)	99 (36.5)	93 (35.2)
750 mg	3 (1.0)	39 (14.4)	39 (14.8)
Total	291 (100.0)	271 (100.0)	264 (100.0)

Some patients experienced repeated dose changes, therefore, these numbers diverge from the number of patients with/without changes.*Total* refers to those patients where dosing information was available

At baseline, 290 patients (99.3%) reported the use of cardiovascular medication other than ranolazine, and 156 patients (53.4%) received additional concomitant medication for the treatment of any comorbidities (Table 3). In total over 50 different medications have been listed to treat a wide range of different comorbidities in participating patients. During the study, changes in concomitant cardiovascular medications were reported for 46 patients (15.9%), and changes in general concomitant medication for 4 patients (1.4%). The most frequent medications comprised acetylsalicylic acid (169 patients; 57.9%), bisoprolol (118; 40.4%), glyceryl trinitrate (93; 31.9%), simvastatin (83; 28.4%), nicorandil (77; 26.4%) and atorvastatin (75; 25.7%). All other medications were prescribed to <25% of the patients. Itemized to ATC2, antianginal drugs were by far the most commonly used medication (336 prescriptions comprising 22 different drugs to 115.1% of the patients), followed by beta blockers (225 prescriptions comprising 20 drugs; 77.05%) and renin angiotensin aldosteron system (RAAS) inhibitors (200 prescriptions of 25 drugs; 68.49%). Of note 47 patients received CCB, of those 35 in combination with beta blockers. While acetylsalicylic acid was the most frequently mentioned co-medication, other antiplatelet therapies have been used in 49 patients. Of those, 23 patients combined acetylsalicylic acid and other antiplatelet therapies and 32 patients were treated with anticoagulants.

**Table 3:** Concomitant medication itemized to substance classes

Medication class	Number of prescriptions *n* (%)	Number of drugs
RAAS inhibitors	200 (68.5)	25
Ca antagonists	52 (17.8)	9
Beta blocker	225 (77.1)	20
Antihypertensive combination	35 (12.0)	19
Diuretics	65 (22.3)	17
Antianginal drugs	336 (115.1)	22
Antithrombotics	141 (48.3)	10
Other medications	496 (169.9)	134
Total	1550 (530.8)	256

*RAAS* Renin angiotensin aldosteron system

### Treatment outcome

**Fig. 1:**
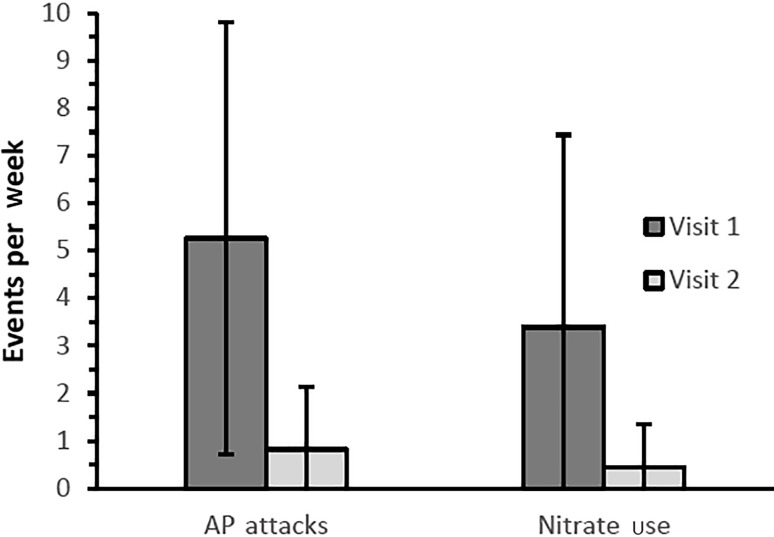
AP attacks and nitrate use per week reported at both visits (arithmetic mean ± SD)

**Fig. 2:**
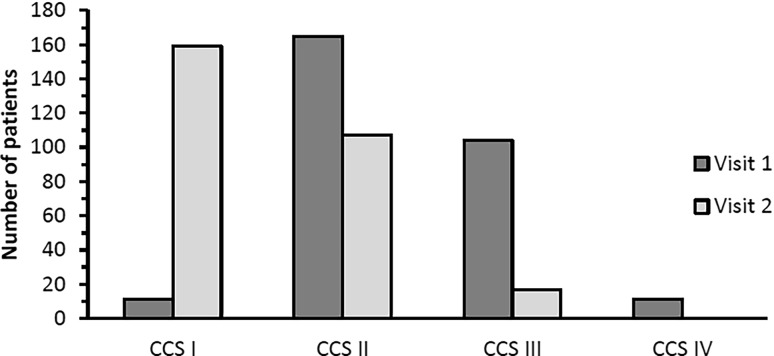
CCS grading in the course of the two visits

During the study period of 12 weeks, the number of angina attacks decreased significantly from 5.3 ± 4.5 to 0.8 ± 1.3 per week (*p* < 0.001). The frequency of nitrate use was used as a second measure for disease activity and a significant decrease from 3.4 ± 4.1 to 0.4 ± 0.9 applications per week was found (Fig. 1). An improvement of exercise capacity and a reduction of AP symptoms was reported by 266 patients (94.0% and 93.7%, respectively). Reduction in disease impact was confirmed by changes in CCS grading (Fig. 2). At visit 1, the majority of patients (269, 92.1%) were classified as CCS II and III, and 11 (3.8%) as CCS IV. At visit 2, no patient was assessed as CCS IV, and the majority of 159 patients (56.2%) as CCS I.

**Fig. 3:**
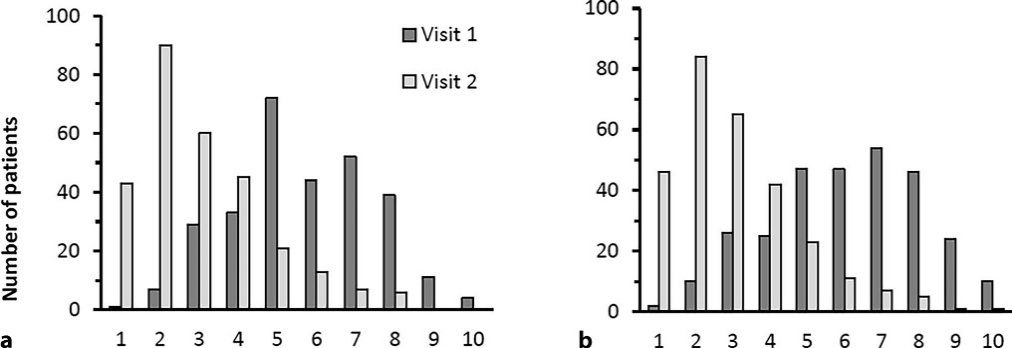
Quality of life at both visits on a Likert scale from 1–10 scale classified by physician (**a**) and patient (**b**)

Heart rate, systolic and diastolic blood pressure showed a trend towards reduction. These changes were statistically significant (not shown) but not assessed as clinically relevant (Table 4). Further changes from visit 1 to visit 2 on several scales were reflected by an increase of QoL, by both physicians and patients (Fig. 3) by using a 10 (poor QoL)—1 (excellent QoL) scale. At visit 1, the average assessment was 5.7 ± 1.8 by physicians and 6.1 ± 2.0 by patients. At visit 2, this rating had improved to 3.0 ± 1.7, as equally assessed by physicians and patients. (*p* < 0.001 for both physicians and patients).

**Table 4:** Vital signs in the course of both visits

	Visit 1			Visit 2		
	Heart rate (bpm)	Systolic blood pressure (mm Hg)	Diastolic blood pressure (mm Hg)	Heart rate (bpm)	Systolic blood pressure (mm Hg)	Diastolic blood pressure (mm Hg)
Min	47	100	50	42	100	56
Max	108	180	120	99	164	108
Median	72	138	80	70	130	80
Mean	72.5	137.3	80.8	69.5	130.7	78.7
SD	11.6	16.1	10.3	9.5	12.4	8.0
*N*	290	292	292	286	285	285

A post hoc analysis revealed limited differences between various subgroups based on demographic or disease characteristics (Table 5). Particular benefits were experienced by the subgroup with potential atrial fibrillation (*n* = 33), as estimated by the use of anticoagulants in patients who presented with arrhythmia in the medical history, where the number of angina attacks per week dropped by 7.7, compared to the subgroup without atrial fibrillation (*n* = 256), where the decrease was 4.0. This difference was highly significant (*p* = 0.005). The same was true for the subgroup with type 2 diabetes mellitus (92 patients, 31.5%), where the decrease was 5.5 attacks per week, compared to 4.1 in the subgroup without diabetes (*p* = 0.002).

**Table 5:** Treatment outcome in various subgroups assessed by reduction (∆) in AP attacks per week

Subgroups	∆ AP attacks	*N* ^a^		
Male vs. female	−4.5 vs. −4.4	161	vs.	125
Diagnosis <1 year vs. ≥1 year	−4.1 vs. −4.8	123	vs.	160
Meteorosensitivity present vs. absent	−4.9 vs. −4.1	115	vs.	174
Beta blocker and CCB vs. beta blocker or CCB	−3.9 vs. −4.5	46	vs.	196
CAD ^b^, macrovascular vs. microvascular disease	−4.7 vs. −4.3	97	vs.	192
Atrial fibrillation ^c^ probable vs. improbable	−7.7 vs. −4.0	33	vs.	256
Type 2 diabetes mellitus, present vs. absent	−5.5 vs. −4.1	92	vs.	183

^a^ Number refer to those patients, for whom data from both visits were available

^b^ Diagnosis made by angiography, administration of statins and aggregation inhibitors

^c^ Indicated by administration of oral anticoagulants.

*AP* angina pectoris, *CCB* Calcium channel blocker, *CAD* Coronary artery disease

### Safety

During the study period five ADRs were reported affecting 3 patients (1.04%). None of these events was serious or clearly related to ranolazine (Table 6).

**Table 6:** ADRs^a^ reported in the course of ARETHA AT

Patient	Original Description	SOCs^b^	Intensity	Relationship
1	Occasional reflux gastritis	Gastrointestinal disorders	Mild	Possible
2	Nausea	Gastrointestinal disorders	Moderate	Probable
2	Vertigo	Ear and labyrinth disorders	Moderate	Probable
3	Liver function levels raised	Investigations	Mild	Possible
3	Renal function tests raised	Investigations	Mild	Possible

^a^*ADR* adverse drug reaction

^b^*SOC* system organ classification

## Discussion

### Limitations of the study

As a non-interventional study, ARETHA AT exhibits specific limitations, including:

- The study was not conducted in a controlled environment; no randomization was performed, no control or placebo group was present and the patients were exposed to a multitude of other therapeutic procedures. Therefore, the observed improvements are not necessarily related to the administration of ranolazine; however, concomitant cardiovascular therapies were constant in 84.1% of the patients. Thus, uncontrolled external factors seem to exhibit only limited influence.
- 292 participants represent 40.6% of the maximum number of 720 patients; however, this number was not determined by formal sample size estimation and is therefore irrelevant for the descriptive character of the results. The 292 patients are roughly equal to 0.1% of 250,000 patients in Austria suffering from stable AP [8].
- Subgroups were formed post hoc, recruitment did not aim at specific proportions for each subgroup; however, the results of the subgroup comparisons are in good accordance with the available literature.
- In the subgroup with patients with atrial fibrillation the diagnosis was not confirmed by an ECG.
- Participants were recruited at practices for primary care and by internal specialists, not at hospitals. Thus, extremely severe cases might have been underrepresented.
- There was no standardized test to measure the improvement in exercise capacity.

### Generalizability

The ARETHA AT study focused on the situation of patients with stable angina in Austria. Demographic features were in good accordance with the results of a recent registry [6]. Austrian patients were not included in ranolazine registration studies and ARETHA AT patients differed by inclusion and exclusion criteria from the restrictions of controlled studies. Patients from all major regions of Austria were recruited in ARETHA AT, therefore reducing the risk of geographical bias. In addition, the outcome data showed high levels of internal consistency and were in accordance with studies from other European countries, such as OSCAR [9], ARETHA DE [10] and CARISA [11]. In contrast to these studies, a high percentage of female patients were recruited which is a major strength of ARETHA AT, since most other approaches are significantly biased in favor of male participants. The ARETHA AT study captured data which had not been considered consistently in the studies cited, including CCS grading, QoL estimates, trigger factors, comorbidities, methodical aspects of the first diagnosis, thus achieving a highly integrated view on the situation of the patients. These data may exclusively represent the situation in Austria, since no comparison is possible yet; however, these results are in accordance with observations from a different geographical region, the Commonwealth of Independent Nations [12]. Thus, a considerably degree of generalizability as well as validity of data can be assumed in spite of all weaknesses unavoidably connected with non-interventional studies. Furthermore, the ARETHA AT study considered the subjective well-being of the patients in addition to clinical endpoints, such as reduction in angina attacks and use of nitrates, and found consistent evidence for improved QoL. This kind of evidence was rarely addressed for other treatment options [13–15].

### Interpretation

Both the EMA (European Medicines Agency) approval and the applicable guidelines [2, 4] recommend the use of ranolazine for stable AP patients with inefficient first-line treatment. In accordance with these guidelines, the participating physicians usually prescribed ranolazine in order to improve exercise capacity, reduce symptoms, and reduce angina despite other treatments. Although ranolazine treatment usually starts with the recommended dosage, many patients are not uptitrated but are given the starting dose throughout the treatment. Even without an increase in dose, a more pronounced improvement was found compared to CARISA [11], comparable to the results of ARETHA DE [10, 16]. The observational study OSCAR [9] achieved a higher percentage of patients reaching CCS I; however, the longer observation period of OSCAR compared to ARETHA AT potentially explains most of the differences.

It might be suggested that this improvement in spite of the omitted uptitration indicates that the observed changes were not causally related to ranolazine, in accordance to some early and highly critical reviews on ranolazine [17]. Ranolazine, however, was prescribed only if first-line treatment had failed or were not applicable. Thus, the consistent improvement found by ARETHA AT in spite of predominantly unchanged concomitant medication strongly suggests a causal relationship as the best explanation of these real-life observations. The effectiveness of ranolazine was further confirmed by comparison with studies on alternative treatment strategies. An analysis on the use of trimetazidine for the treatment of stable angina pectoris [18] resulted in a significant improvement of numerous scales as well but the reduction of angina attacks per week (−1.3–−0,6 vs. −4.5 for ranolazine) and nitrate use per week (−1.4– −0.5 to 2.3 vs. −3.0 for ranolazine) were less pronounced compared to ARETHA AT. Ivabradine seems to be most effective in patients with a heart rate exceeding 70 bpm [14, 19], which were found in 50% of the ARETHA AT population. In addition, some important and highly debated trials [20–23] cast doubts on the prognostic impact of invasive revascularization as first-line therapy and underline the importance of optimizing medicinal treatment. Thus, the results of ARETHA AT may support the suggestion [24] that ranolazine should become an integral part of optimal medicinal treatment of CAD, especially due to its compatibility with many other drugs, even dronedarone.

Although some of the subgroups studied by ARETHA AT were relatively small, the usability of ranolazine for a wide range of patients was confirmed. Females were only poorly represented in previous studies [11]. The results of the ARETHA AT study suggest that ranolazine is equally suitable for both genders (compare also [25]). This finding is of specific relevance since the prevalence of a severely impaired coronary flow reserve leads to a higher mortality in female AP patients [26]. In patients with atrial fibrillation (as indicated by administration of oral anticoagulants), the reduction in AP attacks was far more pronounced than in other patients (−7.7 vs. −4.0). This finding is in accordance with previous studies [27, 28] and confirms atrial fibrillation as another comorbidity where ranolazine may be suitable. Furthermore, AP attacks decreased significantly more in the subgroup with type 2 diabetes mellitus than in other patients (−5.5 vs. −4.1), in good accordance with other real-world data [10]. As shown in previous studies, ranolazine seems to reduce glycated hemoglobin (HbA1C) levels, thereby exerting a positive effect on diabetes itself [29–31]. Treatment of patients with angina and type 2 diabetes mellitus is considered as challenging [10], therefore supporting the use of ranolazine in patients with CAD and diabetes.

The safety of the treatment was confirmed by a low number of ADRs and a low number of early withdrawals or discontinuations. Within approximately 12 weeks, ranolazine was withdrawn in 4.5%, compared with, e. g., 22.1% within 8 weeks of nicorandil [15]. No deterioration of vital signs was found but heart rate was lowered and systolic and diastolic blood pressure showed a trend towards improvement. These results have to be interpreted with caution because no standard method was defined in the protocol to measure the blood pressure and because the cardiovascular co-medication was changed in almost 16% of patients with a wide range of co-morbidities and co-medications. Ranolazine is not known to affect hemodynamic parameters based on large controlled studies [32] (a study with aberrant results [33] used intravenous administration and is therefore hardly comparable); therefore, adaptations in concomitant medication or life style may be likely reasons for the small changes in hemodymanic parameters. A tendency towards lowering of blood pressure was observed during the trial, although it may have been influenced by other concomitant medication. Thus, the safety profile of ranolazine summarized in the recent literature [10, 24, 34] is confirmed, and ranolazine can be considered as an important symptomatic therapy of CAD [34]. Subgroup analyses suggest that ranolazine can be used safely in patients with a wide range of comorbidities.

## Conclusion

- Ranolazine uptitration as possible by the SmPC is not a frequent treatment option for Austrian patients; however, patients still exhibit improvement.
- Patients exposed to ranolazine experience improved quality of life.
- In patients with stable angina and concomitant type 2diabetes mellitus or potential atrial fibrillation a specific benefit of ranolazine may be expected.
- Ranolazine is equally suitable for both genders.
- Ranolazine is tolerable and carries a favorable safety profile with a low number of ADRs when used according to Austrian real-world standards
